# WormRACER: Robust Analysis by Computer-Enhanced Recording

**DOI:** 10.1007/s11357-025-01631-8

**Published:** 2025-03-26

**Authors:** Bennett T. Van Camp, Quinn N. Zapata, Sean P. Curran

**Affiliations:** 1 Leonard Davis School of Gerontology, University of Southern California, Los Angeles, USA; 2 Redmond, USA

**Keywords:** *C. elegans*, Movement, Tracking, Software, Analysis

## Abstract

The pace of scientific high-throughput screening for age-related phenotypes requires the need for developing streamlined and efficacious methods of measuring and quantifying physiological outcomes and at a scale that enables adequate statistical power to measure the variation in populations. Here, we introduce Worm Robust Analysis by Computer-Enhanced Recording (WormRACER), a computationally efficient computer vision software capable of extracting six different crawling and swimming metrics from many animals simultaneously, including worm area, worm length, crawling speed, swimming speed, dynamic amplitude, and wave initiation rate (thrashing). Additionally, we developed a web-based portal that provides metric averages and metric vs time graphs that allow for simple data analysis and quality assurance. WormRACER will facilitate the rapid and quantitative characterization of movement as a facile measurement of healthspan enabling power for high-throughput screening of genetic, environmental, and pharmacological interventions.

**Supplementary Information:**

The online version contains supplementary material available at 10.1007/s11357-025-01631-8.

## Introduction

Animal behavior is nuanced and varied [1], which makes documentation and analysis challenging at the level of an individual, but exponentially complex at the population level. The study of animal behavior led to a better understanding of how animals perceive and respond to environmental changes. In the past, these studies and observations were manually recorded and analyzed [2], which required significant time and effort, but at times lacked the accuracy needed to define relevant differences between models. However, advances in documentation technologies [3] have allowed for the automation of these recordings and calculations as well as an increase in the depth of characterizable phenotypes, such as reverse swimming [3], but importantly that also enable higher throughput screens that minimize human error and bias [4].

*Caenorhabditis elegans*, which displays a rich and complex set of behaviors despite only containing 302 neurons [5, 6] is an example of a research model that has benefitted from the development of new technologies to track movement and behavior [7, 8]. These new tools have advanced drug screening, behavioral studies, and more detailed quantification of physiological changes [9–13] and as such there is a significant and continued demand for enhancing high-throughput measurements and tracking of *C. elegans* behavior to advance the field.

One field of *C. elegans* research that has benefitted greatly from these advances in behavior tracking is aging biology [3, 13–16]. With age, mobility decreases in both *C. elegans* and humans [3, 17–19]. Additionally, mobility is a key factor in the quality of life in older adults [20]. Thus, the ability to accurately and efficiently track behavioral phenotypes is imperative to the study of aging in *C. elegans*.

Several movement trackers have been developed that can analyze multiple *C. elegans* behavioral responses to genetic, environmental, and pharmacological interventions [3, 4, 21–31]. Each of these platforms accounts for multiple variables, but each documentation system can integrate a different number of data points and importantly prioritizes these readings differentially which can limit the scope of each experiment and often imposes limits on the speed of data analysis. Although several parameters are important, several universally important considerations for *C. elegans* research include:

1. Number of trackable animals: Trackers opt to either follow a single worm [32] or many, even hundreds, of worms simultaneously [3]. While tracking multiple worms allows for more efficient data collection, single-worm trackers often also include a moveable stage that can shift to prevent the worm from leaving the field of view [32] which enables longer recordings and data collection and analysis.
2. Environments: These are the surroundings the animals are placed in during measurement. Commonly used environments include substrate plates where animals freely explore [7], liquid media (M9, NGM) where animals swim [3], and, more recently, microfluidics devices which restrict movement, but tissue movements (e.g., rhythmic pumping of the pharynx) are measured [31].
3. Collision tracking: Collisions are a limiting factor when it comes to multi-worm trackers [7]. In standard computer vision-based trackers, worms cannot be tracked during a collision which hampers the amount of viable data points that are generated [7]. Some trackers have made significant strides in tracking worms through collisions using a geometric worm model, though this can often increase processing time [7].
4. Software and hardware dependencies: The recording and computational abilities and capacity directly influence the capabilities, and the cost of tracking systems, particularly if environmental chambers that maintain temperature, humidity, and light are needed and if high-resolution optics (e.g., microscope objectives and cameras) are required for the study. Similarly, the length of recordings impacts the storage capacity needs of the system, and the speed of computational analysis is dependent on the processing power which each comes with added cost. Some worm trackers opt to use third-party software such as MATLAB [28] for data analysis. Additionally, the use of more computationally intensive methods, including AI and machine learning, demands higher computational capacity to analyze recordings in order to maintain similar processing speeds [33].

In consideration of these variables, here we report the development of WormRACER, a user-friendly worm tracker with a focus on both a low cost-to-entry and high processing speed for users with varied resources. That makes this technology universally accessible. WormRACER is capable of identifying, tracking, and analyzing the movements of multiple individual animals on both solid media and in liquid environments and on a variety of microscopes. We demonstrate that WormRACER can rapidly, efficiently, and accurately analyze data acquired with microscope-mounted cameras. Finally, a web-based version of WormRACER for server-based processing eliminates the dependency on expensive hardware to analyze data, which collectively reduces the barrier to entry for this technology.

## Design and implementation

### Algorithm

**Fig. 1:**
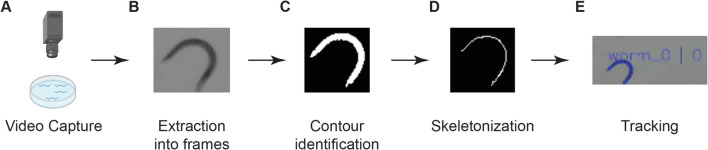
WormRACER workflow. After the video capture and upload to WormRACER (**A**), the program begins by separating the video into frames and analyzing them in chunks (**B**). For each frame, the image is preprocessed, and the outline of each worm is identified (**C**). The midline of these outlines, or contours, is then found in a process called skeletonization (**D**). Using the midpoint of the midline and the overall contour of each worm, WormRACER then tracks each worm through the video (**E**). It then returns a video that shows the tracking of each worm along with Excel tables for each tracking metric, both frame by frame and an overall average (Figs. 2 and S1)

**Fig. 2:**
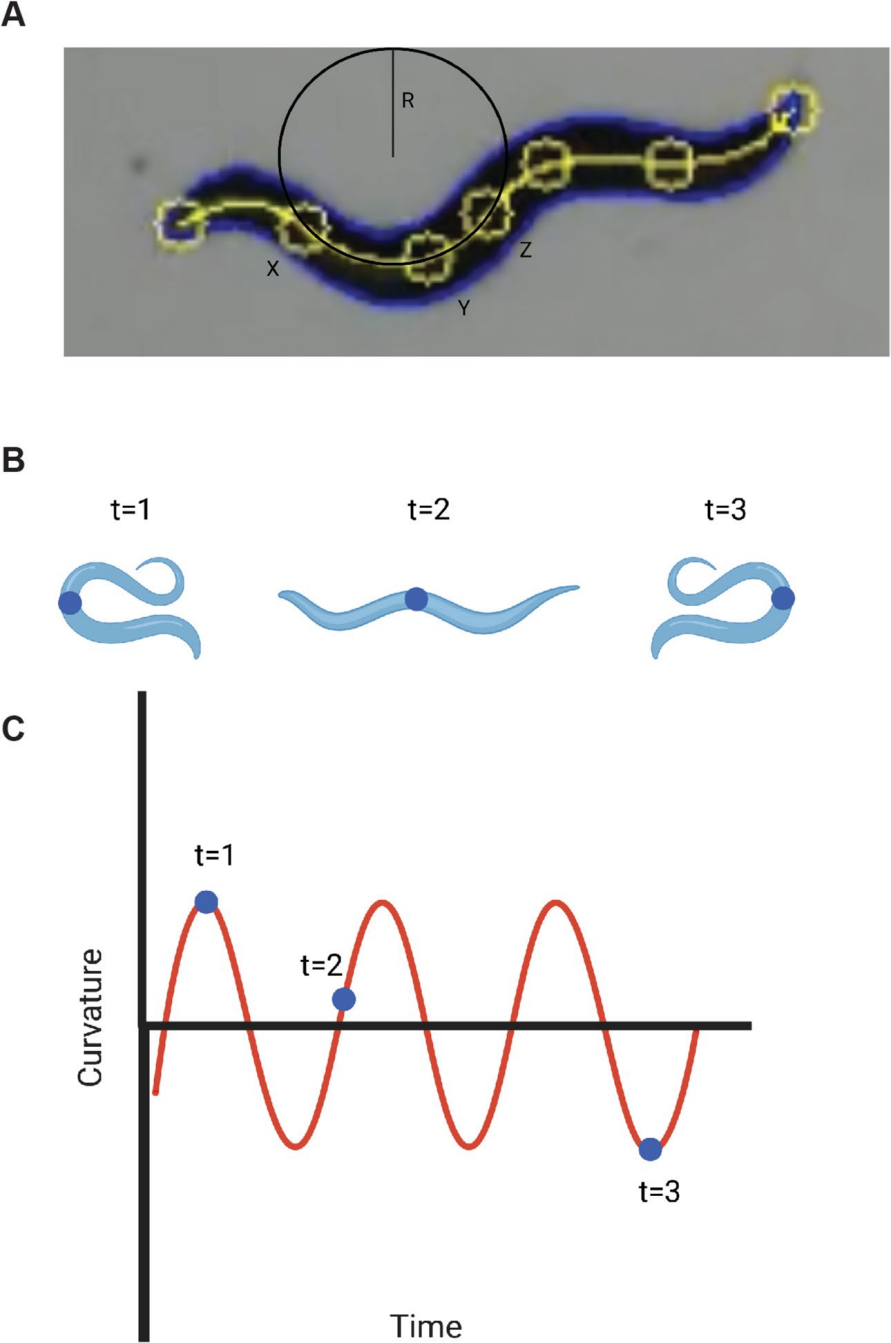
WormRACER movement analysis. **A** The worm area is the area inside the blue outline. Worm length is the length of the yellow midline. Worm speed and swim speed are calculated using the position of the midpoint (labeled *Z*). Curvature is calculated at *Y* as 1/*R* of the circle that overlaps points *X*, *Y*, and *Z*. This is calculated for each point shown on the midline, except for the ends. For wave initiation rate, a wave happens every two times the curvature of point *X* crosses zero. Point *Z* is the midpoint of the worm. (**B**) Diagram of a worm in different orientations at different times and (**C**) diagram illustrating a curvature vs. time graph for the midpoint of the worm diagramed in (**B**)

The WormRACER package includes image analysis software capable of assessing six distinct physiological metrics, including worm area, worm length, crawling speed, swimming speed, dynamic amplitude, and wave initiation rate (thrashing), and an online web-based user interface (Fig. S1) that can efficiently analyze videos and automatically graph and group data, allowing for easy visualization and troubleshooting. Once the user has uploaded a video and entered a Worm Minimum Size, Unit Conversion Factor (to convert from pixels to um during calculations), and Frame Percentage value (the minimum percentage of frames a worm must appear in), WormRACER begins by chunking the video and processing each frame in parallel (Fig. 1). WormRACER then removes artifacts from the video and finds the contours (worms) in each frame with size gating based on the Worm Minimum Size [34]. Additional filtering is performed to remove contours that touch the edge of the frame. This data is stored in a thread-safe data structure so that worm tracking and data processing can happen in parallel. This data structure enables WormRACER to track a worm and determine collisions by looking exclusively at contours and by tracking their positions within the thread-safe data structure. Then, each worm is assigned an ID and tracked simultaneously. Lastly, each data metric is calculated from the tracked worms (Fig. 2). While designing WormRacer, 7.5 fps and 14 fps videos were tested, and successful contour tracking was confirmed for both speeds. For each worm, positional and curvature data is stored for multiple points along the skeleton in order to calculate the downstream measures.

In terms of tracking and with respect to worm collisions, previous systems have been developed to track worms through collisions; however, we opt to censor that data [7]. While there are certainly applications for this type of technology, we operated under the premise that, for the measures included here, the data generated during a collision would be inherently confounded for the metrics tracked by WormRACER. This is largely due to the limitation of being unable to perfectly track worms through collisions with the technology currently available. Although previously developed trackers have used geometric worm models to improve collision tracking, there is still a gap in tracking accuracy between worms in a collision and uncollided worms [7]. Therefore, we focused instead on detecting collisions, suspending data collection during the collision, resuming data collection after the worms separate, and assigning each worm a new identification (ID) number. While this could hinder the number of ID events, we anecdotally found that a moderate number of worms (20–50) could be utilized, with a minimum frame percentage of 50% with at least 200 total frames of data.

### Measures

WormRACER provides data for six different commonly used parameters: worm area, worm length, crawl speed, wave initiation rate, swim speed, and dynamic amplitude. These metrics are defined similarly to previous worm tracking systems and are described in brief below [3]. These metrics were also chosen as they have been associated with the age-related decline in mobility [3, 13, 14]. Specifically, crawl speed, wave initiation rate, and swim speed decrease with age/decreased muscle function while dynamic amplitude increases with age [3, 13, 14]. Together, these metrics aim to provide a coherent analysis of worm health via movement analysis.

1. Worm Area is determined by multiplying the number of pixels within the detected contour of a worm by the Unit Conversion Factor squared (Fig. 2A). This factor is determined via a photo of a ruler with the camera in the same position as when the video was initially taken. Put simply, the Worm Area is determined by counting the pixels of the worm and then converting that number into µm^2^.
  $$Worm Area=Worm Pixels*{\left(Unit Conversion Factor\right)}^{2}$$
2. Worm Length is characterized by thinning [35], a method that processes the contour of a worm to find the midline, and multiplying the number of pixels by the Unit Conversion Factor (Fig. 2A). In other words, worm length is determined by counting the pixels of a worm’s midline and converting that number to um.
  $$Worm Length=Worm Midline Pixels*Unit Conversion Factor$$
3. Crawl Speed is calculated by taking the distance the midpoint of a worm, Point* Z* in Fig. 2A, has traveled divided by the time difference between each frame (Fig. 2A). The midpoint of a worm is defined as the middle pixel along the worm’s midline. Put another way, worms are simplified down to a single point, their midpoint, and tracked through each frame. The distance the midpoint travels between each frame is divided by the change in time between each frame to get the Crawl Speed.
  $$Crawl Speed=\left(Midpoint Position 1-Midpoint Position 0\right)*Unit Conversion Factor*Video Frame Rate$$
4. Wave Initiation Rate is the number of body waves created per minute. This is tracked by creating seven points along the skeleton of the worm and recording the curvature of the worm at each point using Menger’s Curvature [36] (Fig. 2B). Put another way, the curvature is defined as the inverse radius of the circle of best fit at each tracking point (Fig. 2A). A stroke is counted whenever the curvature of the end of the worm crosses zero in either direction. A wave is then counted every two strokes. Notably, all strokes are counted and do not separate the directionality or sign of the waves. Lastly, partial strokes are not counted and are rounded down to the last complete stroke.
  $$Curvature at y=c\left(x,y,z\right)=1/R$$
5. Swim Speed is similar to Crawl Speed but the calculation is performed in a two-stroke interval to minimize lateral movement contributions in a comparable manner to previously developed trackers [3] (Fig. 2A). This is because worms will jitter laterally when they stroke. However, that movement is not necessarily representative of the movement of the worm as it is canceled out immediately when the worm initiates its next stroke in the opposite direction. To minimize these contributions, the position of the worm is only taken every two strokes when measuring swim speed. This would be akin to measuring how fast a runner is moving on the belt of a treadmill and omitting the lateral movement of their head bobbing as they run.
6. Dynamic Amplitude is defined as the maximum difference in curvature in the worm found during a given two-stroke interval (Fig. 2A). Of note, the maximum and minimum curvatures do not have to come from the same tracking point along the worm. In short, Dynamic Amplitude is the measure of the magnitude of the curviness of a worm’s movement with a higher Dynamic Amplitude indicating a greater curve.
  $$Dynamic Amplitude=\left|Maximum Curvature\right|-|Minimum Curvature|$$

## Results

The utility of worm tracking software can differ based on the number of animals that can be tracked simultaneously, the types of trackable environments (e.g., plates, liquid), collision tracking capabilities, and other software/hardware dependencies (MATLAB or expensive computer setups) [3, 4, 7, 21–32]. WormRACER was specifically designed to provide a computationally streamlined worm tracker that could track the most common metrics such as crawl speed and wave initiation rate with minimal, if any, differences compared to commercially available products. Indeed, WormRACER can run using only a microscope-mounted camera and any computer with an internet connection.

**Fig. 3:**
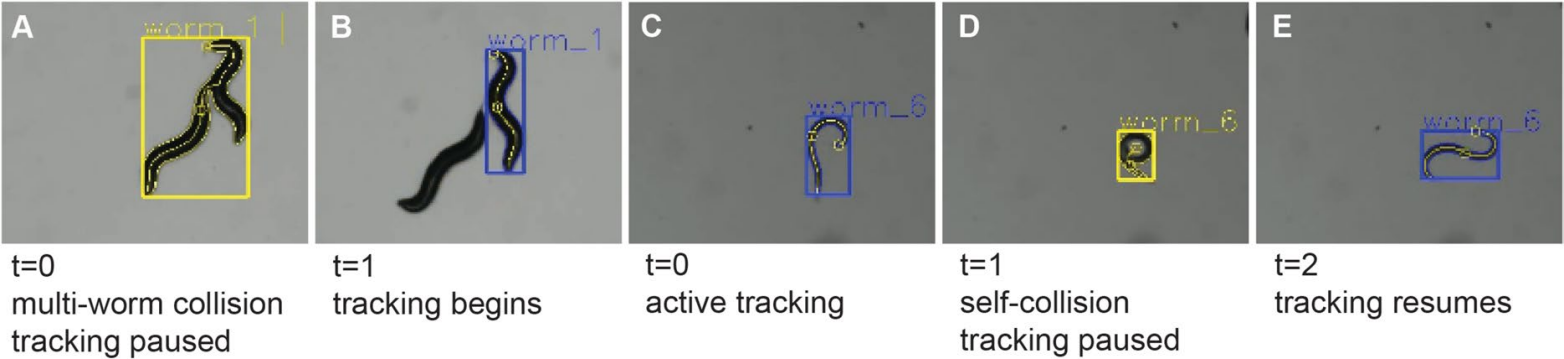
WormRACER collision detection. Examples of collision detection and resolution. **A** During a multi-worm collision, data collection does not occur until the worms separate completely. Then, tracking begins. **B** When a worm collides with itself or curls, data collection pauses until the worm exits the self-collision

One significant choice in the development of WormRACER was the decision not to track worms through collisions with another object (Fig. 3A–B) or with self (Fig. 3C–E). Instead, when the outline animals intersect, measurement of these animals is suspended until the collision is resolved (Fig. 3). Collisions are documented (yellow designation, Fig. 3A and D ) as this data can be useful in specific contexts, such as studying grouping, complex turns, or mating behavior, collisions could act, but since this can confound the measures, we in WormRACER (e.g., wave initiation rate, dynamic amplitude, and speed) tracking does not resume until individual animals are resolved (blue designation, Fig. 3B and E).

**Fig. 4:**
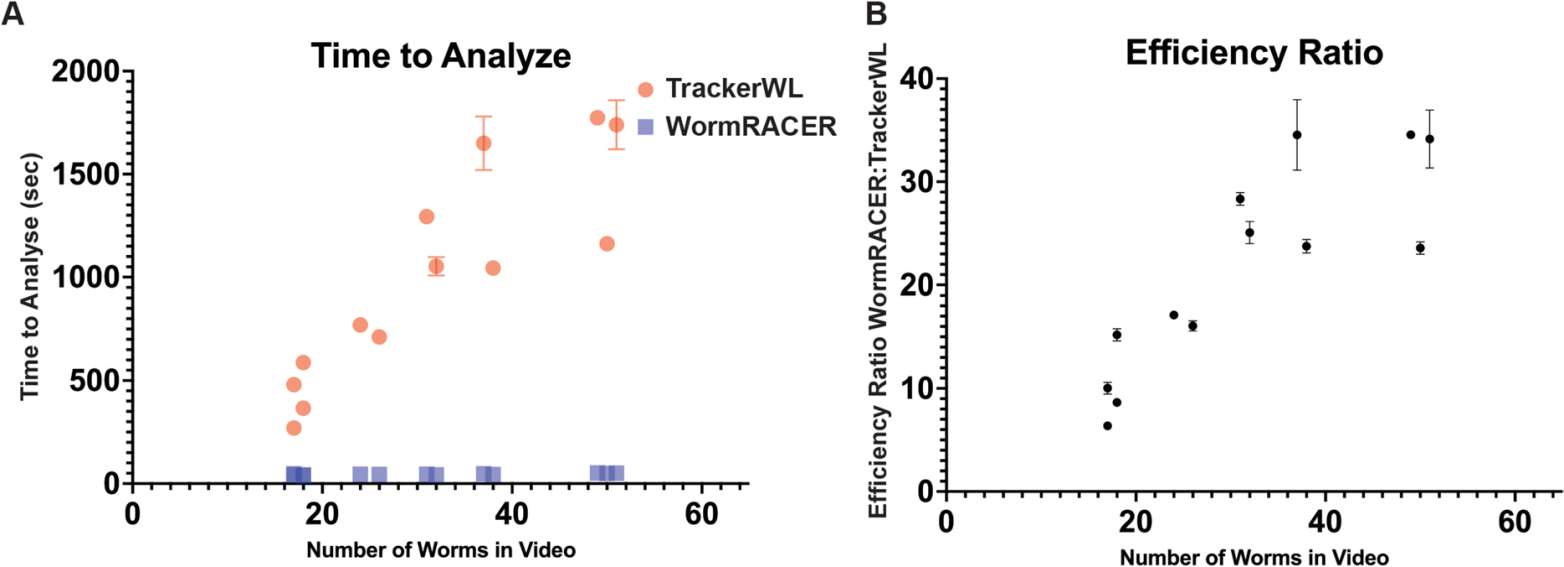
WormRACER efficiency comparisons. Comparisons of analysis time for the crawling and thrashing videos of Day 1 adult worms using in Fig. 2 between WormRACER and Tracker WL. **A** Comparison of raw analysis times. All WormRACER videos took under a minute to analyze. **B** Fold increase in analysis efficiency between WormRACER and Tracker WL. Points without displayed error bars had a variance in analysis time too small to graph

We validated the WormRACER platform using videos of L4 and day 1 adult worms (Videos S1–S2). In our analysis of the accuracy of WormRACER, we compared identical recordings to a commercially available tracker, henceforth referred to as Tracker WL, which we use routinely for all our movement studies [13]. However, it should be noted that WormRACER was developed independently and is not based on Tracker WL’s code. Here, when comparing the local version of WormRACER to Tracker WL, we note a significant-fold increase in analysis speed that increases with the number of animals in the video (Fig. 4A–B). For example, an analysis of a crawling video with 17 worms was ten times faster to complete with WormRACER and a crawling video with 49 worms was 34 times faster to analyze (Fig. 4A). All the videos that were tested took under a minute to analyze with WormRACER (Fig. 4A).

**Fig. 5:**
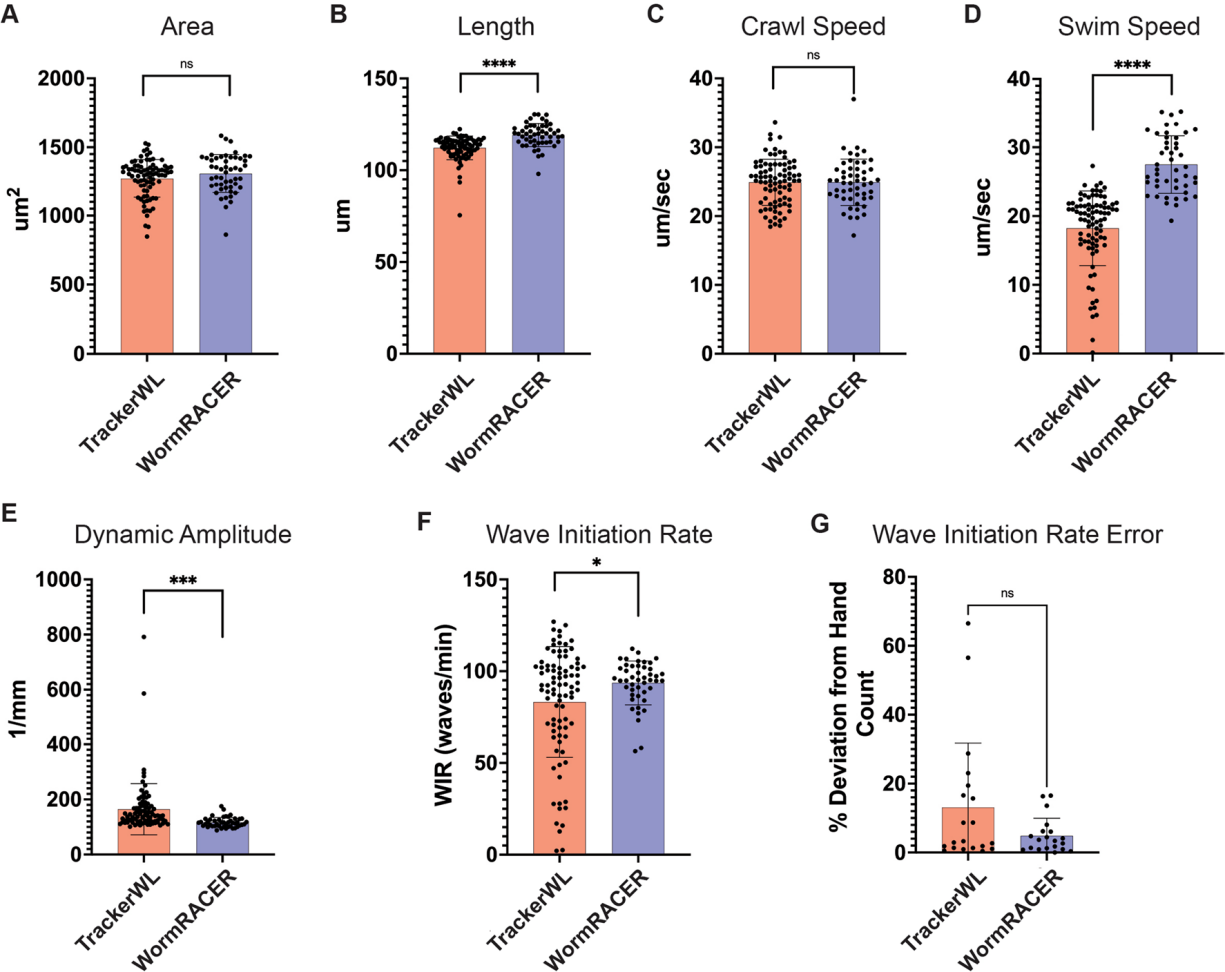
WormRACER comparison analysis. Comparison data between Tracker WL and WormRACER for day 1 adult worms for **A** worm area, **B** worm length, **C** crawl speed, **D** swim speed, **E** dynamic amplitude, **F** wave initiation rate, and **G** wave initiation rate error. For each metric, six videos, two for each biological replicate, of either crawling or thrashing worms were used, each with around 20–50 worms. Crawling videos were used for Worm area, worm length, and crawl speed and thrashing/swimming videos were used for swim speed, dynamic amplitude, and wave initiation rate. All statistics were done using an unpaired *t*-test, *p < 0.05

We note that WormRACER has comparable outcomes for day 1 adult animals in the worm area and crawling speed to Tracker WL (Fig. 5A and C). Additionally, WormRACER has comparable outcomes for L4 stage animals in crawling speed and wave initiation rate to Tracker WL (Fig. S2C and F). We do, however, find subtle differences when comparing worm length, swim speed, dynamic amplitude, and wave initiation rate in day 1 adult worms and for worm area, worm length, swim speed, and dynamic amplitude for L4 stage worms (Fig. 5B and D–F and Fig. S2A, B, D, and E).

Lastly, we validated that WormRACER functions on a variety of microscopes and on aged worms. WormRACER was tested on up to D6 adult worms on a Leica M205 scope and was able to successfully track the worms (Fig. S3).

## Discussion

We created computationally efficient computer vision software that can rapidly quantify six different metrics of *C. elegans* physiology, including worm area, worm length, crawling speed, swimming speed, dynamic amplitude, and wave initiation rate (thrashing). Additionally, WormRACER is able to track multiple worms at once, with the amount only being limited by the field of view of the camera used. The software is designed to be easy to learn, with the goal being to provide a highly accurate and reliable method of automated video analysis. WormRACER requires minimal user input for setting the minimum worm size and the frame percentage cutoff to minimize false positives. Although *C. elegans* movement is used as the data model here, WormRacer could be adapted to analyze video of any moving or growing object thus broadening the utility of this software beyond the current application.

### Advances in C. elegans movement tracking

While many previous systems have been created to quantify *C. elegans* behavior [3, 4, 7, 21–32, 37], WormRACER was developed with the goal of providing a system that can quantify the most commonly used physiological parameters while providing significant gains in terms of speed of analysis and usability (Figs. 4 and 5), which have remained significant barriers to entry. We attribute the difference in analysis time to the differences in the design goals between WormRACER and Tracker WL (Table S1). For example, Tracker WL can track a wider array of phenotypes and provide the benefit of collision tracking that can potentially provide longer recordings of individual animals via their geometric prediction model [7]. However, this approach can be computationally intensive in videos with many collisions. On the other hand, WormRACER was designed to measure the most commonly used metrics using a less computationally intensive computer vision-based approach while eliminating expensive hardware and software dependencies that could limit the financial feasibility of some research groups.

WormRACER can process video frames and worm contours in parallel utilizing thread-safe data structures. Such a degree of parallelization greatly contributes to the analysis speed difference between WormRACER and comparable software. While WormRACER is designed to be accessible on as much hardware as possible, the efficiency of the program scales significantly with the number of threads the host can provide. This approach represents a paradigm shift in the worm tracker space, as WormRACER is the first worm tracker to make use of this technology [3, 4, 21–31].

As for the minor differences found between WormRACER and Tracker WL, for the metrics of swim speed, dynamic amplitude, and wave initiation rate, these differences are expected and the result of the design choice to censor collision frames as this censor frames where a worm would be moving slow and be highly curved (Fig. 5D–F and Figs. S2D and E). Additionally, while we find no overall difference in the deviation from hand-counted wave initiation rate between WormRACER and Tracker WL, we do note that the Tracker WL worms with the highest deviation are all worms that collide with themselves or other worms, further supporting the idea there are further iterations that could be made in collision tracking and that censoring collision data is appropriate based on these limitations (Fig. 5G). For the metrics of worm area and length, these are expected as Tracker WL leaves off the ends of the worm where there are zero or one inflection points [3, 7] whereas WormRACER does not (Fig. 5B and Figs. S2A and B).

### Accessibility and future directions

WormRACER was developed in C ++ and is accessible via a web browser for quick and easy-to-use accessibility by the research community (Supplemental Document 1). One of the benefits of this approach over other available tracking methods [7, 37] is a decrease in the required user input. For WormRACER, the user only has to upload the video and enter in a “unit conversion factor” and desired “frame percentage”. Additionally, WormRACER includes metrics vs. time graphs upon completion of analysis to ease troubleshooting and the ability to bulk download analyzed data in one Excel file. Overall, we provide the next iteration in worm behavior analysis with software that can efficiently analyze copious amounts of worms and requires minimal training to use due to the easy-to-use web-based graphical user interface. WormRACER was validated using videos of day 6 (D6) adult, day 1 (D1) adult, and larval stage 4 (L4) worms and on a variety of microscopes; noting that the D1 adult and L4 videos and their comparisons to Tracker WL were restricted to the MBF microscope since Tracker WL is optimized for that platform. Other developmental stages can be used with WormRACER, provided adequate contrast is recorded between individual animals and the substrate at the desired magnification. WormRACER is also able to track many animals individually and for extended durations with a much smaller increase in the required analysis time and computer hardware requirements. By being able to analyze standard, minute-long, videos (20–50 worms) faster than they can be taken, WormRACER eliminates the technological bottleneck that can restrict experimental throughput. Additionally, while WormRACER is optimized for the analysis of *C. elegans* movement, it is a code base that could be adapted to analyze the two-dimensional movement of other subjects as well, such as rodents, flies, tardigrades, yeast/bacteria colony growth rates, or any entity in a two-dimensional space. Lastly, WormRACER removes the need for expensive, high-end computer hardware and only requires a microscope-mounted camera setup and any computer with an internet connection. Moreover, future versions of WormRACER can be updated to accept videos taken on a standard phone camera, thereby eliminating the need for an expensive camera setup. By removing the financial and technological barriers, WormRACER is ideal for research studies but can also be applied to field research and laboratory-based teaching applications.

## Materials and methods

### C. elegans strains and maintenance

All strains were grown at 20 °C on a 6-cm nematode growth media (NGM) + streptomycin plates seeded with OP50. All worms were unstarved for at least three generations before use. Wild type (WT), N2 Bristol strain was the only strain used in this study.

### Movement measurements

For all assays, worms were egg-prepped and synchronized overnight as L1s. Worms were then added to an OP50 plate and allowed to grow for 48 h for L4 assays and 72 h for Day 1 adults. Worms were then washed with M9 + triton onto an unseeded NGM plate. The worms were allowed to acclimate for 30 + min to allow the liquid to evaporate. Videos of worms crawling were recorded for 1 min at 7.5 fps. One milliliter of M9 was then added to the plate, and the worms were acclimated for a minute. Videos of worms thrashing were recorded for 30 s at 14 fps. All imaging was done with the MBF Bioscience WormLab microscope for day 1 (D1) and larval stage 4 (L4) videos. For day 6 (D6), a Leica M205 scope was used. Analysis was then performed either by WormRACER or Wormlab (Tracker WL) version 2023.

### Benchmarking

All benchmarking was performed on a desktop computer running 64-bit Windows 10 Education version 22H2 with 16 GB of RAM and an AMD Ryzen 5 3600X 6-Core Processor 3.80 GHz. Tracker WL analysis was done on Windows while WormRACER analysis was done on a Windows subsystem for Linux since there is no operating system that can run both software. Benchmarking was done after restarting the computer and with nothing else open. Videos were tested sequentially (not multiple iterations on the same video back-to-back) per iteration to prevent caching of video files. All videos were analyzed in triplicate.

### Statistical analysis

All statistical analysis was done on GraphPad Prism version 9.5.0.

## Conclusion

In summary, WormRACER provides the next iteration in *C. elegans* tracking that focuses on reducing the time required to analyze data. WormRACER can analyze videos in under a minute, which represents up to thirty-four times increase in efficiency compared to other analysis platforms and without a decrease in accuracy. WormRACER also comes with the option to censor data during worm collisions to remove inherently confounded movement data. By removing the efficiency barrier and the need for expensive computer hardware, WormRACER is a powerful new resource tool for research and education settings that require high-throughput and high-precision analysis of *C. elegans* behavioral dynamics.

## Supplementary Information

Below is the link to the electronic supplementary material.

Supplementary file1 (DOCX 755 KB)

Supplementary file2 (AVI 42.5 MB)

Supplementary file3 (AVI 41.8 MB KB)

## Data Availability

All data for this paper are within the text and its supplementary materials.
