# Supplementary material for: WormRACER: Robust Analysis by Computer-Enhanced Recording
Source: GeroScience. 2025 Mar 26;47(3):5377–87. doi: 10.1007/s11357-025-01631-8 (PMC12181165; doi:10.1007/s11357-025-01631-8)
Supplement: Supplementary file 1 — Supplementary file1 (DOCX 755 KB) [file 11357_2025_1631_MOESM1_ESM.docx]

**SUPPLEMENTAL MATERIALS**

1. WormRACER Protocol
2. Supplemental Figure S1
3. Supplemental Figure S2
4. Supplemental Figure S3
5. Supplemental Table S1
6. List of Supplemental Videos

**WormRACER Protocol**

Video Recommendations:

- Crawling: 60 second video with at least 7.5 fps
- Thrashing: 30 second video with at least 14 fps
- Videos should not include the edges of the plate and should include minimal debris

Usage:

1. To upload a video, click the three bars in the upper left-hand corner then click upload video.
   1.
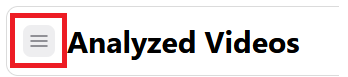

   2.
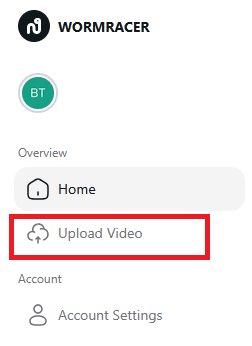

   3. Once uploaded the video will show in a pending state on the homepage until analysis is complete. Uploading multiple videos as once will cause them to run in parallel which will slow down individual processing time but complete all videos around the same time.
      1. For L4 videos we recommend using the following settings:
         1. Minimum Worm Size: 75
         2. Frame Percentage: 50
         3. Frame Start/End: 0,0 (Defaults to analyzing the whole video)
      2. For D1 videos we recommend using the following settings:
         1. Minimum Worm Size: 300
         2. Frame Percentage: 50
         3. Frame Start/End: 0,0 (Defaults to analyzing the whole video)
2. Returning to Home will now display your video and indicate when the analysis is finished. Clicking on the video title will show that videos data (including the video of worm tracking and data graphs)
   1.
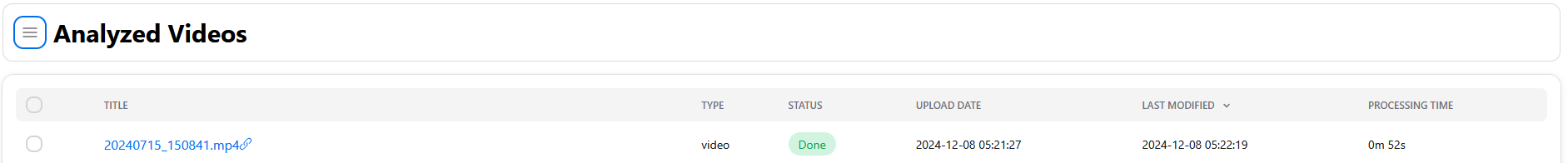

3. Hit Download Data to download excel files and analyzed video. The data is ready to be graphed in the software of your choice.
   1.
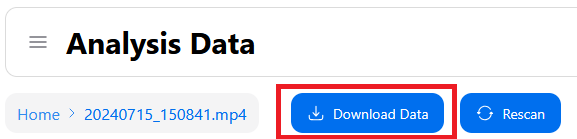


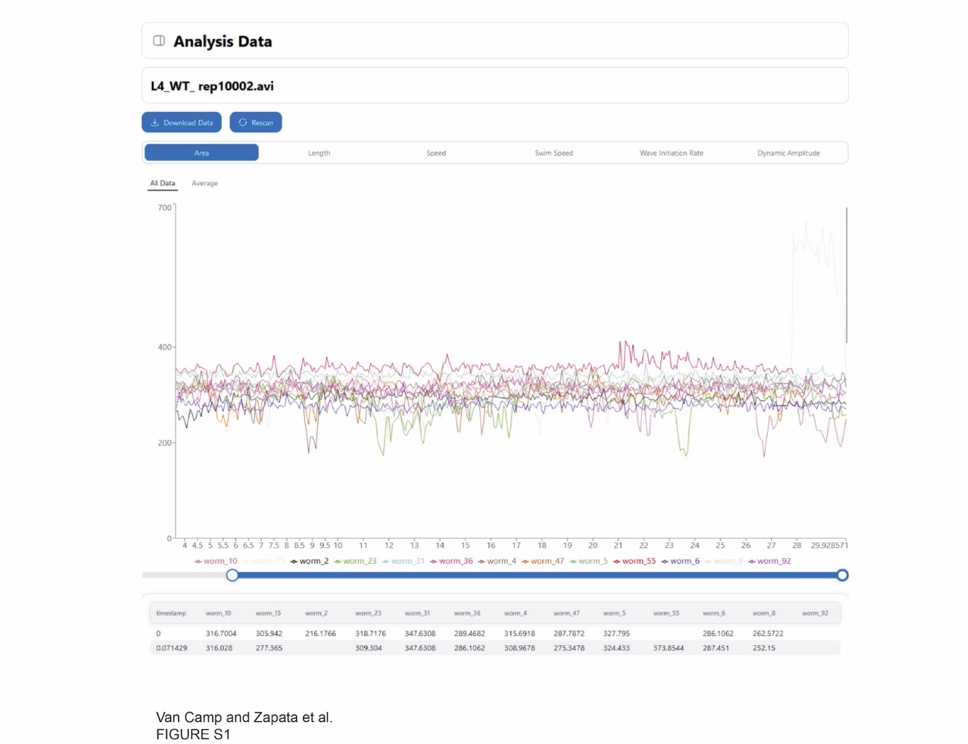


**Figure S1:** The web interface provides a graph tracking each worm throughout the video (area tracking shown). It additionally allows the user to select what time frame of the video is graphed using the blue slider below the graph. Lastly, the web interface allows for the bulk download of data for multiple analyzed videos at once.


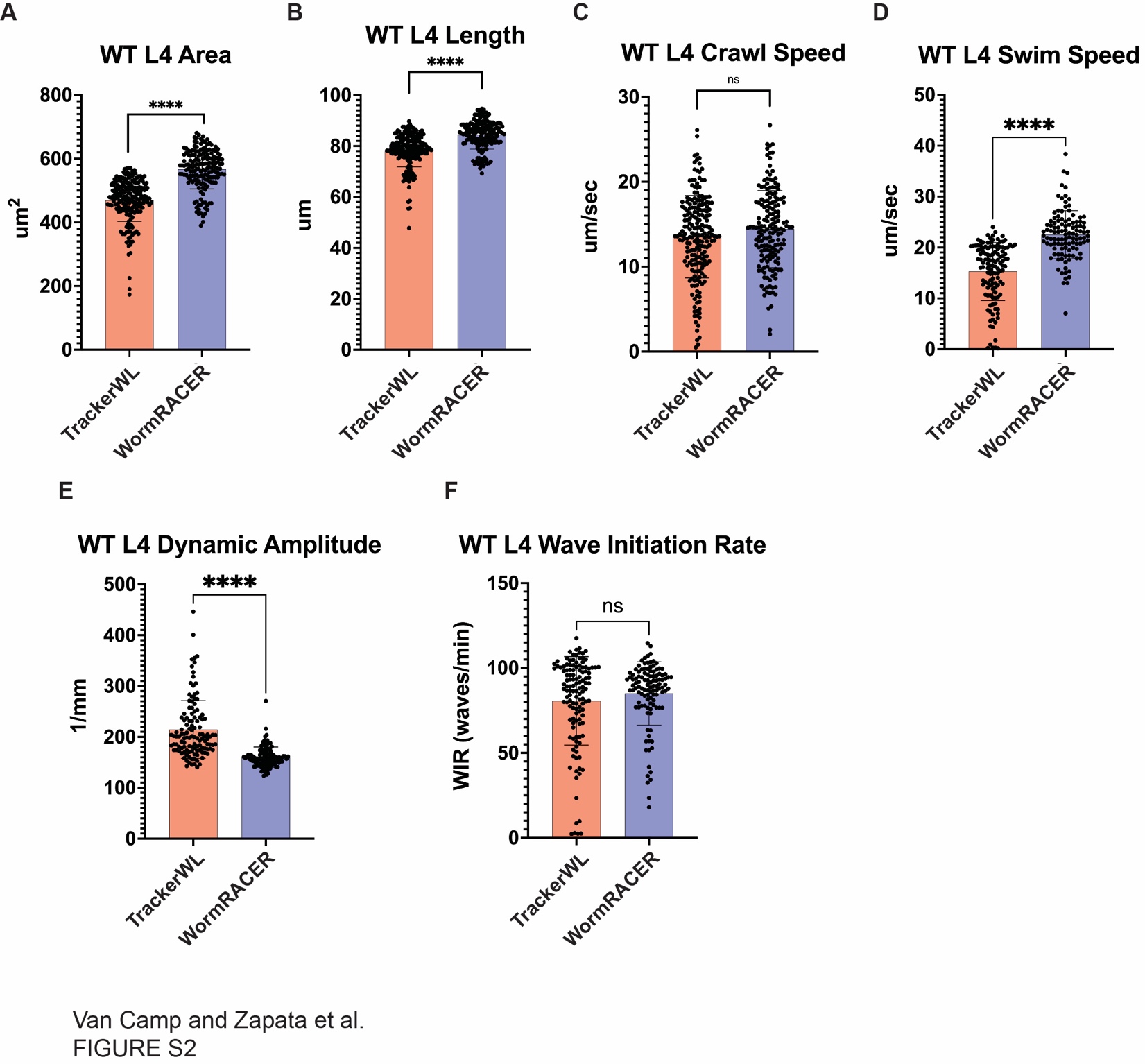


**Figure S2:** Comparison data between Tracker WL and WormRACER for L4 stage worms for **(A)** Worm Area, **(B)** Worm Length **(C)** Crawl Speed, **(D)** Swim Speed, **(E)** Dynamic Amplitude, **(F)** Wave Initiation Rate, and **(G)** Wave Initiation Rate Error. For each metric, six videos, two for each biological replicate, of either crawling or thrashing worms were used, each with around 20-50 worms. Crawling videos were used for Worm Area, Worm Length, and Crawl Speed and thrashing/swimming videos were used for Swim Speed, Dynamic Amplitude, and Wave Initiation Rate. All statistics were done using an unpaired t-test, * means p<.05.


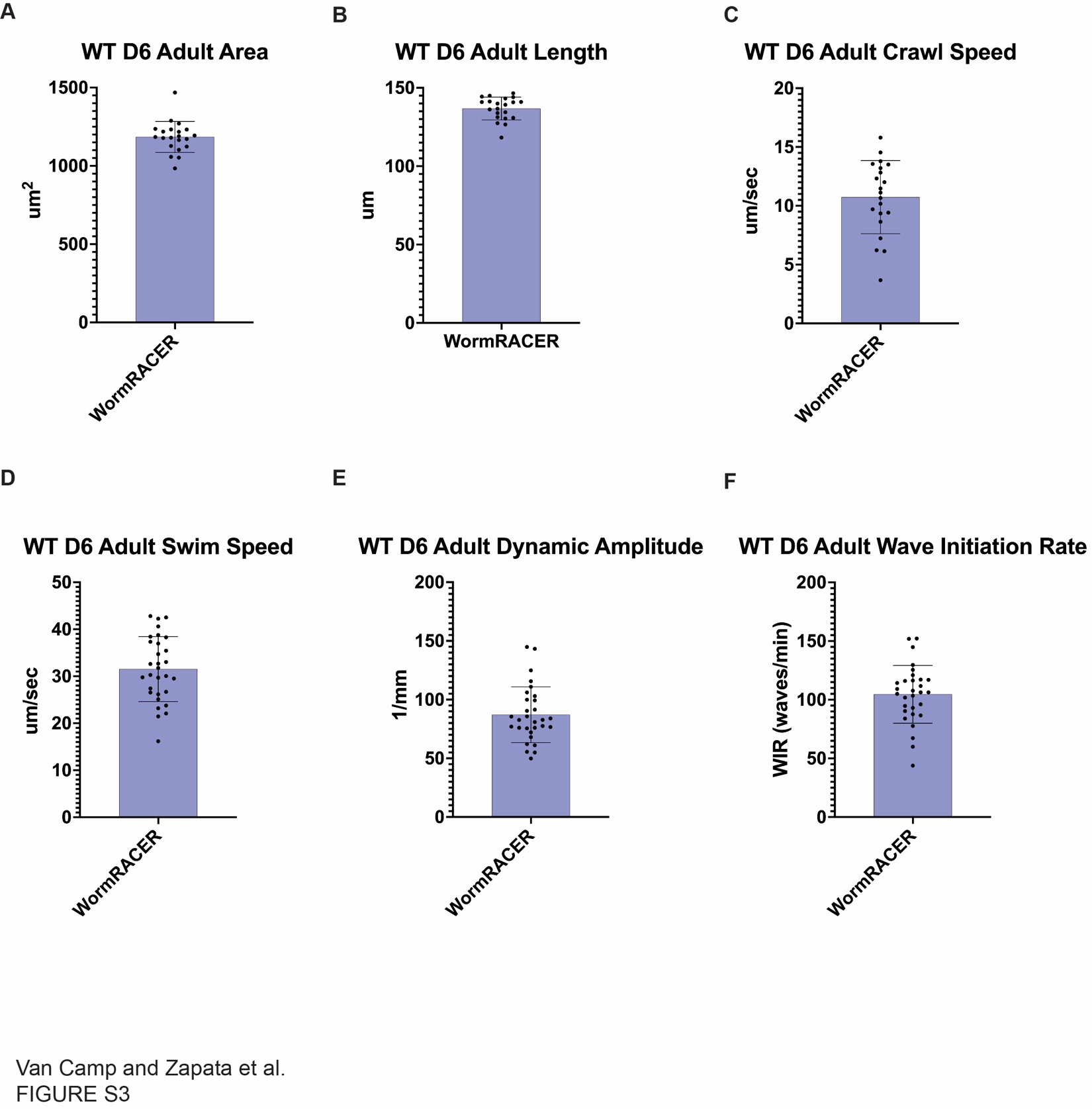


**Figure S3:** WormRACER data for Day 6 adults worms for **(A)** Worm Area, **(B)** Worm Length **(C)** Crawl Speed, **(D)** Swim Speed, **(E)** Dynamic Amplitude, **(F)** Wave Initiation Rate, and **(G)** Wave Initiation Rate Error. For each metric, four videos, each a technical replicate, of either crawling or thrashing worms were used, each with around 10-20 worms. Crawling videos were used for Worm Area, Worm Length, and Crawl Speed and thrashing/swimming videos were used for Swim Speed, Dynamic Amplitude, and Wave Initiation Rate. These videos were taken on a Leica M205 scope.

|  | **WormRACER** | **Tracker WL** |
| --- | --- | --- |
| **Number of Trackable Animals** | Multiple | Multiple |
| **Environments** | Substrate plates, liquid media | Substrate plates, liquid media |
| **Collosion Tracking** | Collision censorship | Yes |
| **Analysis Speed** | Under a minute for standard videos | Up to almost 30 minutes for standard videos (depending on the number of worms and collisions) |
| **Purpose** | Quickly tracking common worm metrics | Tracking complex worm metrics and tracking through collisions |

Table S1: Comparison table of the various features and applications of WormRACER and Tracker WL.

**SUPPLEMENTAL VIDEOS**

Video S1: Example video of a Day 1 Adult crawling video used to make Figure 3.

Video S2: Example video of a Day 1 Adult thrashing video used to make Figure 3.
